# Coupling Planar Cell Polarity Signaling to Morphogenesis

**DOI:** 10.1100/tsw.2002.105

**Published:** 2002-02-15

**Authors:** Jeffrey D. Axelrod, Helen McNeill

**Affiliations:** Department of Pathology, L235, Stanford University School of Medicine, Stanford, CA 94305-5324, USA; Developmental Patterning Laboratory, ICRF, 44 Lincoln’s Inn Fields, London WC2A 3PX, UK

**Keywords:** planar cell polarity, tissue polarity, morphogenesis, Frizzled, signaling, cytoskeleton, Usher syndrome, convergent extension

## Abstract

Epithelial cells and other groups of cells acquire a polarity orthogonal to their apical–basal axes, referred to as Planar Cell Polarity (PCP). The process by which these cells become polarized requires a signaling pathway using Frizzled as a receptor. Responding cells sense cues from their environment that provide directional information, and they translate this information into cellular asymmetry. Most of what is known about PCP derives from studies in the fruit fly, *Drosophila*. We review what is known about how cells translate an unknown signal into asymmetric cytoskeletal reorganization. We then discuss how the vertebrate processes of convergent extension and cochlear hair-cell development may relate to *Drosophila* PCP signaling.

## INTRODUCTION

Epithelial cells are polarized along their apical–basal axes, allowing specialization of function on their apical and basolateral surfaces[1,2]. Cues for polarization along these axes are derived from direct contacts with other cells and the substratum (reviewed in [3]). Many epithelia are also polarized along an axis orthogonal to the apical–basal axis, resulting in asymmetry within the plane of the epithelium[1,2,4,5,6,7] (referred to as Planar Cell Polarity [PCP] or tissue polarity). Polarization along this axis requires a signal to reach across vast expanses of cells. The identity of this signal is not clear at present. However, a signaling mechanism regulating PCP has been partially characterized in *Drosophila* epithelia, and recent results suggest that this mechanism may be conserved in vertebrates. The signal generates molecular asymmetry within target cells and then converts this asymmetry to cytoskeletal reorganization. Here, we review our current understanding of the links between the polarized signal transduction apparatus and modulation of

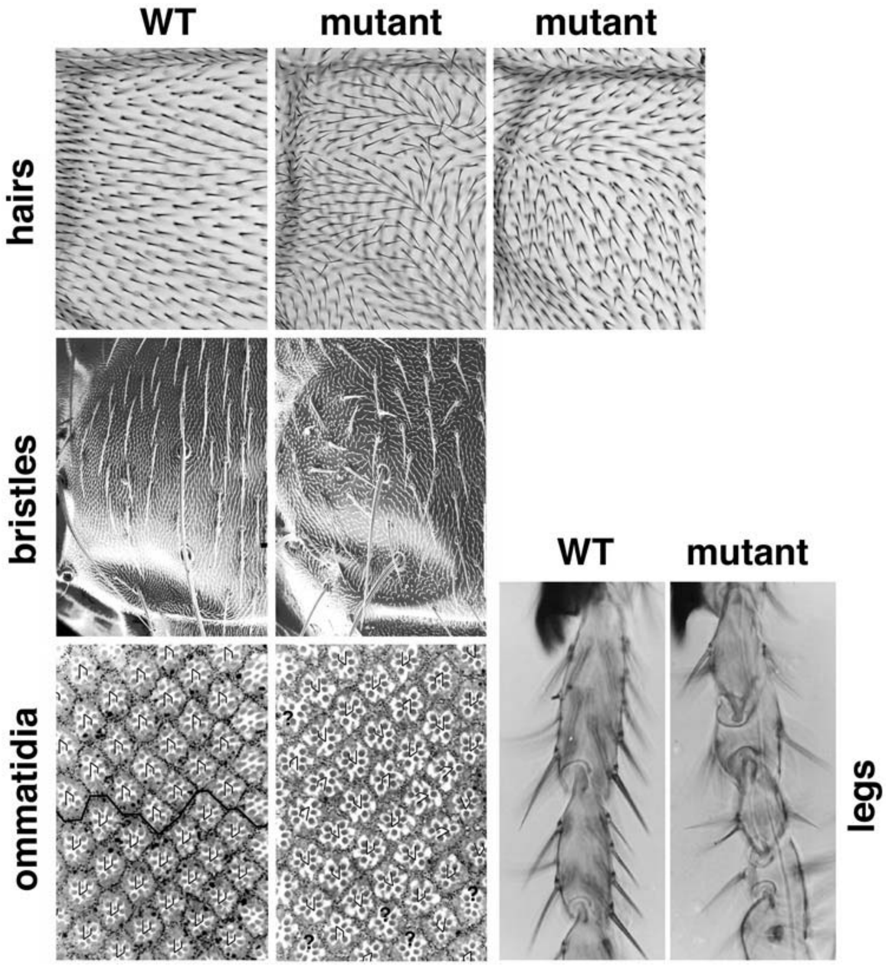

**FIGURE 1**. Phenotypes associated with disruption of PCP signaling. Hairs on epidermal cells (shown on the wing) and bristles (shown on the notum) are in parallel arrays in the wild type. In mutants, hairs and bristles adopt nonparallel orientations, displaying stereotypical swirls and patterns. Some mutants produce multiple hairs from single cells. Ommatidia in the eye are chiral and oriented, with the hemispheres adopting mirror-image asymmetries. This pattern is disrupted in mutants. Wild-type distal legs have tarsi separated by joints. Some PCP mutants produce ectopic, inverted joints. (Revised and reprinted from *Trends Genet*. **14**, Shulman, J.M., Perrimon, N., and Axelrod, J.D. Frizzled signaling and the developmental control of cell polarity. pp. 452-458. Copyright 1998, with permission from Elsevier Science.)

the cytoskeleton in *Drosophila* epithelia. We then consider how this mechanism has been conserved in vertebrate development.

In *Drosophila*, a number of adult tissues display planar polarity, exhibiting arrays of asymmetrically organized cellular structures or multicellular developmental units. On the thorax and abdomen, hairs and bristles project posteriorly, and on the wings and legs, these structures point distally. Planar polarity is also apparent in the eye, where each ommatidium possesses an intrinsic polarity and chirality. The planar polarity of these structures is easily recognized by simple observation (Fig. 1). A powerful set of genetic and molecular tools has made *Drosophila* an extremely attractive system for investigating the controls governing PCP. Studies in *Drosophila* have demonstrated that planar polarization is regulated by a signaling pathway utilizing Frizzled (Fz), a seven-transmembrane protein, as a receptor, and Dishevelled (Dsh), a multidomain cytoplasmic protein, as a signal transducer[4,5,6,7]. A complete list of proteins known to participate in PCP signaling or execution is provided in Table 1. Genetic epistasis exp-

**TABLE 1**
**Proteins Known to Participate in PCP Signaling or Execution**

| Gene Name | Symbol | Protein Domains and Function | Ref. |
|---|---|---|---|
| *Atypical protein kinase C* | *aPKC* | Serine/threonine kinase | 60 |
| *Barbu* | *Barbu* | E(spl)-like protein, suppresses Notch signaling | 39 |
| *Bazooka* | *Baz* | Multiple PDZ domains, known as Par3 in *Caenorhabditis elegans* | 60 |
| *Crinkled* | *Ck* | Myosin heavy chain | 17 |
| *Dachsous* | *Ds* | Large, atypical cadherin-like protein | 49, 85 |
| *Diego* | *Dgo* | Ankyrin repeat protein | 13 |
| *Discs large* | *Dlg* | MAGUK protein containing three PDZ repeats, an SH3 domain, and a guanylate kinase domain | 56 |
| *Dishevelled* | *Dsh* | Cytoplasmic protein; DEP, DIX, and PDZ domains | 66, 86 |
| *Expanded* | *Ex* | Tumor suppressor gene with SH3 binding sites and an N-terminal 4.1 homology region | 47, 87 |
| *Fat* | *Ft* | Large, atypical cadherin-like protein; tumor suppressor | 49, 75 |
| *Flamingo/Starry night* | *Fmi/Stan* | Novel seven-transmembrane cell adhesion molecule, cadherin-like | 11, 12 |
| *Four-jointed* | *Fj* | Novel transmembrane protein, may be cleaved and secreted | 88 |
| *Frizzled* | *Fz* | Seven-transmembrane protein, putative receptor for Wnts | 89 |
| *Frizzled2* | *Fz2* | Seven-transmembrane protein, Wingless receptor | 90 |
| *Fuzzy* | *Fy* | Novel protein with four putative transmembrane domains | 91 |
| *Inscuteable* | *Insc* | Novel protein, recruited by Bazooka to apical membrane | 92 |
| *Inturned* | *In* | Novel, putative transmembrane protein | 93 |
| *Janus kinase/ signal transducers and activators of transcription* | *JAK/STAT* | Janus kinases phosphorylate and thereby activate STAT proteins, which then translocate to the nucleus and activate transcription | 34 |
| *Jun N-terminal Kinase (JNK)* | *JNK cascade* | Kinase signaling pathway stimulated by stress-inducing agents and proinflammatory cytokines, terminating in phosphorylation of the transcription factor Jun; elements of the cascade include the Jun-kinase basket | |
| *Mirror,araucan, caupolican* | *Mirr, ara, caup* | Iroquois group transcription factors | 30 |
| *Misshapen* | *Msn* | STE20-like kinase | 44 |
| *Multiple wing hairs* | *Mwh* | Unknown | |
| *Naked Cuticle* | *Nkd* | EF-hand protein, a negative feedback inhibitor of the Wingless pathway | 78 |
| *Nemo* | *Nmo* | Serine/threonine kinase, human homolog localizes to the nucleus | 53, 94, 95 |
| *Numb* | *Numb* | Intracellular protein that inhibits Notch signaling, contains a PTB domain | 56, 96, 97 |
| *Par6* | *Par6* | Cytoplasmic protein, contains a CRIB domain and a PDZ domain | |
| *Partner of Inscuteable* | *Pins* | Binds Inscuteable, contains TPR repeats | 56 |
| *Partner of Numb* | *PON* | Adaptor protein that binds Numb | 56 |
| *PolyADP Ribose polymerase* | *PARP* | Modifies various nuclear proteins by poly(ADP)ribosylation, modifications are dependent on DNA | 48 |
| *Prickle-spiney legs* | *Pk-sple, or Pk* | Novel LIM-domain-containing protein (three isoforms, Pk, M, and Sple, produced from alternatively spliced RNAs) | 37 |
| *Rasputin* | *Rin* | RasGAP SH3-binding protein | 46 |
| *RhoA, Rac, Cdc42* | *RhoA, Rac, Cdc42* | Small Ras-like GTPases involved in remodeling the cytoskeleton | 16, 98 |
| *Rho-associated kinase* | *Drok* | RhoA effector kinase | 17 |
| *Roulette* | *Rlt* | Unknown | 53 |
| *Scribble* | *Scrib* | LAP protein with leucine-rich repeats and PDZ domains | 62 |
| *Spaghetti squash* | *Sqh* | Muscle myosin II regulatory light chain | 17 |
| *Strabismus/ Van Gogh* | *Stbm/Vang* | Putative transmembrane protein with potential PDZ-binding domain | 51, 99 |
| *TGF-β activated kinase 1* | *TAK1* | MAPKKK, implicated in the JNK cascade | 42 |
| *Unpaired* | *Upd* | Ligand for the JAK/STAT pathway, also known as outstretched | 34 |
| *Wingless* | *Wg* | Secreted signaling molecule | 90 |
| *Zipper* | *Zip* | Myosin II heavy chain | 100 |

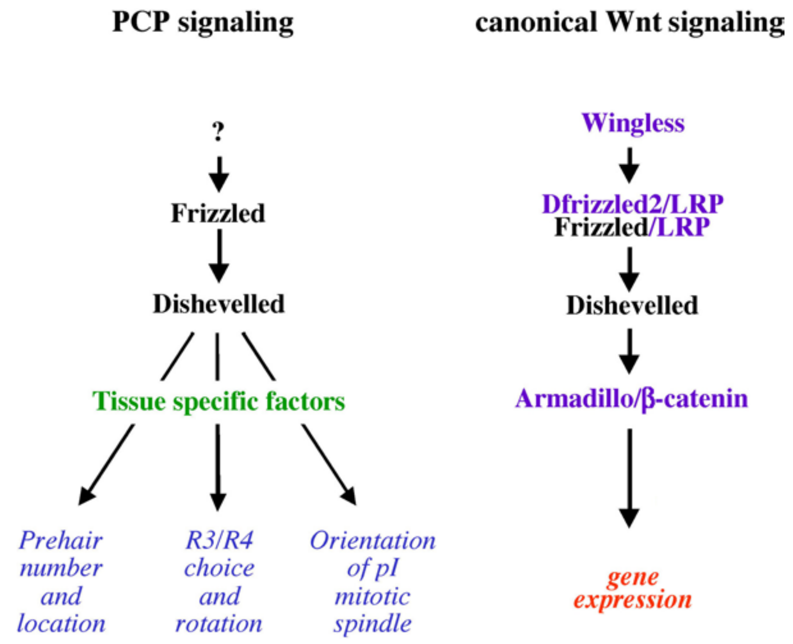

**FIGURE 2**. A simplified schematic diagram of the PCP and Wg (canonical Wnt) signaling pathways. (*Left*) In PCP signaling, Fz likely serves as a receptor for an unknown ligand and acts upstream of the signal transducer, Dsh. In turn, Dsh acts upstream of tissue-specific factors to effect polarity in the various tissues. (*Right*) For comparison, a simplified schematic of the Wg/Wnt signaling pathway is shown. Fz and Dsh function in both pathways. Remarkably, part of the PCP pathway bears close resemblance to the canonical pathway mediating inductive developmental signaling by members of the Wnt family, including *Drosophila* Wingless (Wg, a Wnt-1 ortholog)[101,102,103]. Indeed, Dsh, Fz, and the Fz homologue, DFrizzled2 (DFz2)[104,105,106] are also known to transduce the Wingless signal. However, the downstream components are distinct. The *Drosophila* gene names are shown, though the pathway is conserved.

eriments have suggested an ordering of these components into a PCP signaling pathway (Fig. 2; for a more complete view, see Box 1). The PCP pathway is similar to another well-known developmental signaling pathway mediated by Wingless (Wg)/Wnt-1 (Fig. 2), known as the canonical Wg (or Wnt) pathway. Strikingly, Dsh acts in both the PCP pathway and the canonical Wg signaling pathway. Analysis of Dsh mutations that disrupt PCP signaling but not Wg signaling have demonstrated that there is a specific domain that acts in PCP signaling but not in canonical Wg signaling. The PCP signaling pathway comprises a number of core components whose roles are shared during polarization of the various target tissues, plus additional, tissue-specific components whose roles are more specialized.

**BOX 1. A detailed schematic diagram of the PCP signaling pathway (FIG. A).** The signaling mechanism is understood primarily in genetic terms, though an understanding of the molecular function of some components is beginning to emerge. The components shown in black form the core components and are involved in responding to the global signal by generating subcellular asymmetry. The components shown in green are the tissue-specific factors, whose roles are to read out the subcellular asymmetry and produce cell-type-specific alterations in cell shape and behavior. Many other components whose roles are poorly understood are not shown. Notably, no ligand for Fz has yet been discovered.

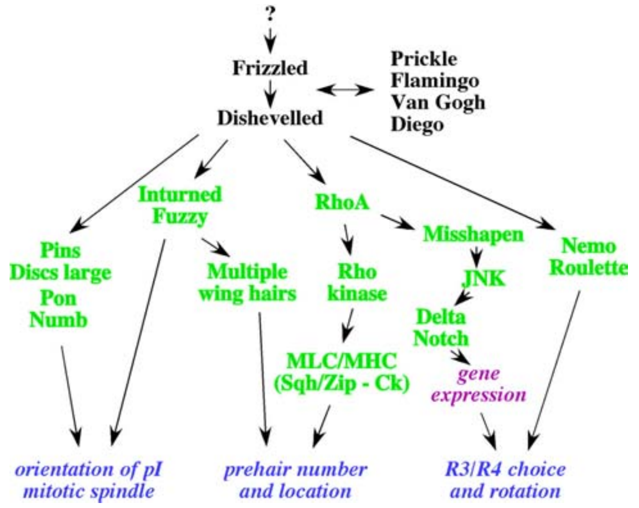

Many of the core components are involved in establishing a molecular asymmetry within target cells, in effect marking one side of the cell in response to a global cue. Recent reports have demonstrated that a Fz signaling complex is assembled symmetrically around cells and then becomes asymmetrically localized during generation of polarity in the pupal wing[8,9,10]. Both Fz and Dsh have been shown to localize to the distal boundary of pupal wing cells, while Flamingo (Fmi), also known as Starry Night (Stan)[11], a cadherin superfamily member, is proposed to localize to both the proximal and distal boundaries[12]. Diego (Dgo), an ankyrin repeat protein, also localizes to these boundaries, though on which side is not known[13]. A feedback loop appears to mediate the acquisition of this asymmetry[8,9]. Mutations that disrupt the asymmetric localization of these components also produce aberrant planar polarity. This correlation strongly suggests that their asymmetric subcellular localization serves as the cue directing cytoskeletal reorganization.

One model is that a similar asymmetric signal will play a role in generating polarity of bristle precursor cells and of ommatidia. The asymmetric signal would be linked, through the action of tissue-specific factors, to elements that control the unique morphogenetic changes observed in each tissue. While the subcellular localization of these proteins have not been reported in the eye, Fmi/Stan was reported not to show asymmetric localization in the bristle precursor cell that is a target of Fz signaling[14]. Furthermore, *Dgo* mutations appear not to affect the polarity of bristles[13]. Therefore, it is not clear whether a similar asymmetric localization of these signaling molecules will play a role in determining the polarity of all tissues.

Critical steps in our understanding of the development of PCP lie in understanding how an asymmetric signal is generated within responding cells, and in uncovering the links between the signal transduction system and the reorganization of the cytoskeleton. In this review, we describe what is known about how this signal links, in different systems, to modulators of the cytoskeleton and cell surface to result in PCP.

## COUPLING ASYMMETRIC SIGNALING TO MORPHOGENESIS IN WING HAIRS

### Cellular Organization

Epithelial cells secreting the adult cuticle each produce a cellular extension called a trichome, or hair. Hairs are oriented in a polarized array, pointing distally on the appendages and posteriorly on the body axis. Planar cell polarity signaling regulates the orientation and the subcellular localization of hairs as well as the number of hairs produced by each cell. Disruption of the signal produces stereotypical swirls and patterns of misoriented hairs and in some cases produces significant numbers of cells with multiple hairs (see Fig. 1 and Box 2 for a description of hair growth).

The actin and microtubule cytoskeletons are dramatically reorganized during PCP signaling in hair cells in the wing. The functional roles of these structural elements also have been examined. Turner and Adler[15] cultured *Drosophila* pupal wings *in vitro* and treated them with microtubule- and actin-destabilizing agents (see Box 3). They were able to conclude that both actin and microtubules are targets of the PCP pathway and that disruption of their function partially phenocopies mutations in some of the PCP genes.

### Known Cytoskeletal Regulators are Targets of the PCP Signal

The small GTPase, RhoA, is a known effector of the actin cytoskeleton. Clones of RhoA mutant cells produce a multiple-hair phenotype and possibly a hair-orientation defect, although these pheno-

**BOX 2. A description of hair growth (FIG. B).** Morphogenesis of hairs has been studied most extensively on the wing[107,108]. Ultrastructural analyses revealed that hairs emerge from the distal aspect of each cell's apex beginning at approximately 33 h after puparium formation (apf) . They are first evident as extensions of the cytoplasm, bounded by membrane, and they contain numerous fibers (consisting of actin; see below) and a few microtubules[20,107]. Hairs are produced by all ~30,000 wing cells, developing in a slightly asynchronous pattern, with distal cells producing hairs before those emerging from more proximal cells[20]. Initially, hairs point distally and lie flat across the apex of the nearest distal neighboring cell. Subsequently, a series of morphogenetic changes occur, during which the cells secrete cuticle, the hairs change shape and assume a more erect posture, and additional fibers and microtubules are deposited within the hair and extend into the main body of the cell. Ultimately, the cell undergoes a dramatic flattening, during which the hair moves from the distal vertex of the cell to near the center, where it emerges from an elevated pedestal on the cell surface[108]. The hairs reach approximately half their adult length over a 16-h period[15].

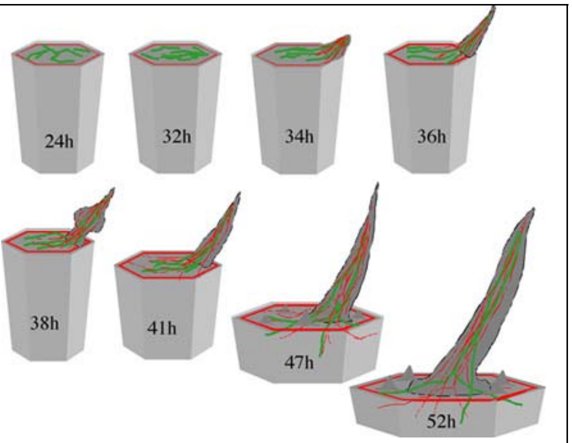

Examination of the actin and microtubule cytoskeletons has produced a correlated picture of the organization of these structural elements. At 24 h apf , the hexagonally shaped apex of each cell contains a microtubule array that is beginning to orient along the proximal–distal axis[15,109]. By 30 h apf, a distinct proximal–distal orientation is evident. During this time, actin is distributed circumferentially around the apex of the cells[15,20,109]. Between 32 and 36 h apf, actin begins to coalesce specifically at the distal vertex of each cell, and shortly thereafter, both the actin and microtubule skeletons begin to extend distally into the nascent prehair. The actin and microtubule networks extend down into the body of the cell, forming a set of "roots" that radiate from the distal vertex[15,20,109]. When the prehair migrates to the center of the cell (between 37 and 46 h apf)[15,107,108], both the actin and microtubule bundles move toward the center. The apical meshwork of microtubules then appears to radiate, like spokes, from the centrally located prehair[15].

Analysis of mutants shows that the regulated steps include localization of the prehair to the distal vertex, and control of the number of prehairs initiated from a single cell. Mutations affecting a "core" set of signaling components[7], including Fz, Dsh, Prickle (Pk), Vang/Stbm, and Fmi/Stan, produce a phenotype in which the prehair, as monitored by actin staining with fluorescent phalloidin, emerges from the center rather than the distal vertex of each cell and points in an aberrant direction ([20,99] and our unpublished observations). These mutants also produce a small number of cells with multiple hairs. Mutation of other components, including Inturned (In), Fuzzy (Fy), Multiple wing hairs(Mwh), RhoA, and Rho-associated kinase (Drok), produce from two to six prehairs that emerge from the periphery of each cell, sometimes in an aberrant direction[16,17,20]. Various arguments place the core components upstream of this latter set of proteins in a signaling pathway[20]. Interestingly, the same core components that regulate the location of prehair emergence are also required to localize Fz and Dsh to the distal boundary of each cell, while the tissue-specific components that regulate the number of prehairs are not needed to localize Fz and Dsh, but rather appear to couple Fz/Dsh localization to the location and number of prehair initiation sites[8,9].

types are not further characterized[16]. Rho has many known effectors, including Rho-associated kinase. Like its vertebrate counterparts, *Drosophila* Rho-associated kinase (Drok) binds specifically to activated Rho and activates by phosphorylation the nonmuscle myosin II (MyoII) regulatory light chain (MLC), encoded by Spaghetti squash (Sqh)[17]. Clones of cells mutant for Drok function display multiple-hair phenotypes as well as ommatidial polarity defects similar to those seen in *rho* mutant clones. Two myosin heavy chains (MHCs), encoded by the *zipper (Zip)* and *crinkled (Ck)* genes, function in conjunction with Rho, Drok, and Sqh (Box 1). While these experiments did not demonstrate that Drok or the downstream components are activated by the Fz/Dsh signal, their activity is clearly required in conjunction with Fz and Dsh to mediate the PCP signal. The involvement of a role for myosins represents the first direct link between the PCP signal and cytoskeletal activity. However, important questions remain unanswered.

The mechanism by which these myosins regulate prehair number is unclear, and the relative roles of the myosin heavy chains Zip and Ck are not understood. Zip, but not Ck, has been shown to physically interact with Sqh, constituting MyoII. However, although phosphorylation of MLC is thought to activate the corresponding MHC, Sqh and Zip modify, in opposite directions, the phenotypes resulting from impairment of Dsh or overexpression of Fz. Furthermore, Ck and Zip, by the same assays, display opposing activities as well. Underlying the difficulty in interpreting these

**BOX 3. Effects of disrupting actin or microtubule polymerization on hair polarity and development**. Developing wings were treated with microtubule- and actin-disrupting agents[15]. Disruption of microtubules with vinblastine produced at least three effects. First, under some conditions, cells in treated wings failed to produce prehairs, demonstrating a requirement for microtubules in this process, as predicted from their presence in hairs. Second, in some cases, cells produced multiple, independent prehairs. These prehairs initiated from the cell periphery, usually in the distal portion of the cell. This phenotype resembles that seen in mutant *in* and *fy* flies. When vinblastine was applied later, a slowing of prehair outgrowth was observed. It therefore appears that the microtubule network, most likely the P–D oriented apical network observed prior to prehair emergence, plays a role in limiting prehair initiation sites at the distal vertex. Later, microtubules are involved in prehair growth. We speculate that the apical, P–D oriented microtubules may be responding to the asymmetrically localized Fz/Dsh complex as a cortical marker, directing the transport of components necessary for prehair formation to the distal vertex of the cell, and that In and Fy may function in this process.

Similar experiments using cytochalasin D produced an overlapping but distinct set of defects. At higher doses, prehairs again failed to emerge. At more modest doses, some cells appeared to initiate multiple prehairs similar to those seen with vinblastine treatment, while others produced branched hairs. Thus, actin fibers are also likely involved in restricting the number and location for prehair emergence, and they are required to maintain the integrity of growing prehairs. Notably, these experiments were unable to phenocopy a significant hair orientation defect such as that seen in *fz* and $dsh^1$ mutants. While it is possible that this reflects little importance for actin and microtubules in determining orientation, it is more likely that it reflects a masking of this role by the pleiotropic nature of the treatments. More generally, this type of experiment would be expected to reveal only a subset of the processes in which actin and microtubules are involved during PCP signaling. Nevertheless, they provide important clues to the mechanisms involved.

results, the genesis of the multiple-hair phenotype is confusing, since both loss of Fz function as well as Fz overexpression are capable of producing it, albeit to different extents. Thus, the finding of a role for myosins gets us a step closer to understanding how prehair initiation is regulated, but additional biochemical and cell biological analyses are required to understand what myosins are doing in this process.

Mutations affecting the Inturned (In), Fuzzy (Fy), and Multiple wing hairs (Mwh) proteins produce multiple-hair-cell phenotypes as well as modest orientation defects[18,19,20]. In and Fy are novel proteins, while Mwh is not yet cloned. These components are likely involved in reading out cellular asymmetry downstream of Fz and Dsh[20], and they probably function in a pathway independent of the RhoA, Drok, myosin pathway[17].

## PLANAR POLARITY IN THE FLY EYE

The fly eye is composed of approximately 800 photoreceptor clusters, called ommatidia, that are arranged into dorsal and ventral fields of mirror-image planar polarity and chirality (reviewed in [21]). Dorsal and ventral fields meet at the midpoint of the eye, which is known as the equator. Each ommatidium is composed of a core of eight photoreceptors, R1–R8, as well as a number of accessory cells. The striking planar polarity of the fly eye is the end product of a dramatic series of cell rearrangements (see Box 4, Fig. E). A wave of differentiation, marked by a constriction of the epithelium known as the morphogenetic furrow, moves from the posterior of the eye disc to the anterior. As cells emerge from the morphogenetic furrow, they are organized into regularly spaced groups of cells, known as preclusters. The preclusters in the dorsal and ventral halves of the eye achieve opposite orientations due to mirror symmetric rotations of the clusters by 90°. The development of planar polarity is clearly more complex in the eye than in the wing and involves changes in cell adhesion and motility as well as a reorganization of the cytoskeleton, and it is correspondingly more difficult to determine how different steps in the pathway are controlled. Although planar polarity in the eye is more complicated than in the wing, it is very clear that the mutations in core PCP components, such as Fz, Dsh, and Fmi/Stan, which perturb PCP in the wing also disrupt PCP in the eye. The process appears to be divided into three distinct steps: establishment of spatial information, interpretation of the signal, and control of rotation.

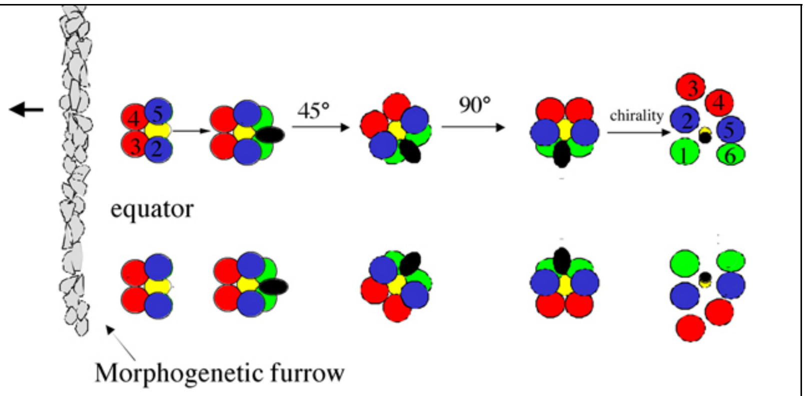

**BOX 4. Development of PCP in the fly eye. FIG. C.** The eye in *Drosophila* develops from an initially unpatterned epithelium, known as the eye imaginal disc. Differentiation starts at the posterior of the eye disc and progresses anteriorly, marked by an indentation in the epithelium known as the morphogenetic furrow. Cells become organized into clusters shortly after emerging from the morphogenetic furrow. Prior to the five-cell cluster stage (which is the first stage shown in this diagram), cells are organized into other arrangements that may be important in the development of polarity[21,36], but for simplicity they are not shown in this Figure. Additional photoreceptors are added to the cluster as it matures, and the cluster begins to rotate. The direction of rotation depends on the D/V position in the eye. Later, rhabdomeres in R3 and R4 undergo cell-specific changes, which result in the appearance of different chiral forms in the dorsal and ventral halves of the adult eye. Anterior is to the left, and progressively more mature forms are shown as they approach the posterior.

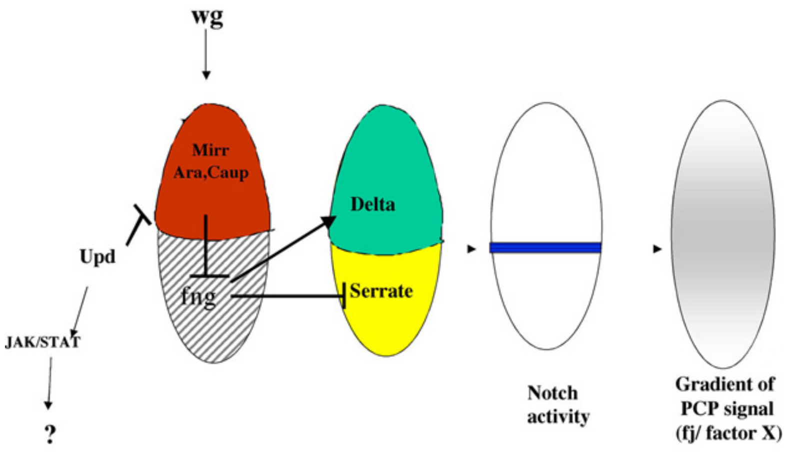

**FIG. D.** The establishment of spatial information in the eye takes place in at least three steps. First, Wg and Upd act to induce *mirror*, *ara,* and *caup* (red). Mirror, Ara, and Caup repress *fringe* expression, restricting it to the ventral half of the eye. Fringe glycosylates Notch, enhancing its ability to signal via Delta and suppressing its ability to signal via Serrate. This results in a narrow band of Notch activity at the midpoint of the eye. Notch in turn induces the expression of Fj, and possibly factor X, which may be expressed in a gradient (grey). Note that this is a simplified version of the expressions of Delta and Serrate, focusing on their role in forming the D/V midline (see [28,29,32]).

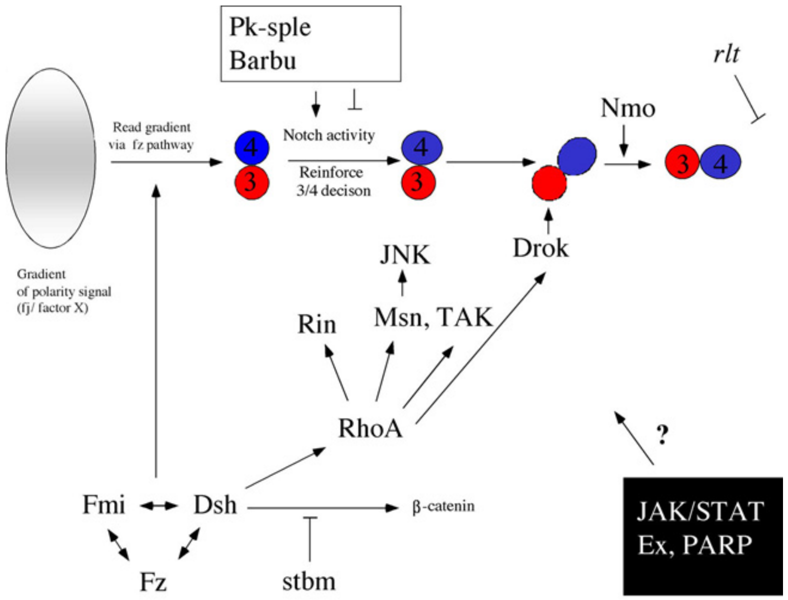

**FIG. E.** Interpretation of the PCP signal and control of rotation. Ommatidial clusters first form, then rotate to 45°, where they briefly pause and then go on to rotate to 90°. Precluster cells are assumed to be relatively equivalent prior to receiving the PCP signal. A gradient of Fj/factor X is interpreted via Fz-PCP pathway. This results in greater activity in the presumptive R3 cell, which is closest to the equator. The Notch pathway then amplifies the differences between the R3 and R4 cells. Finally, the R3 and R4 cells interpret the PCP signal and direct cluster movements. Nemo acts to promote cluster movement from 45° to 90°, while Roulette stops clusters from rotating >90°. Where possible, the figure indicates where the different PCP genes act. Genes for which this is not clear are in a "black box". At least two genes are thought to impact on the regulation of Notch activity and are in the white box.

## Establishing Spatial Information

An initial step in determining polarity in the fly eye appears to be the establishment of spatial information throughout the eye field (Box 4, Fig. D). Wg, acting through the canonical pathway, induces the dorsal expression of the Iroquois family transcription factors *mirror*, *araucan*, and *caupolican* [22,23,24,25,26,27]. Mirror, Araucan, and Caupolican suppress *fringe* expression, thus limiting it to the ventral half of the eye[28,29,30,31]. Fringe is a glycosyltransferase that modifies Notch, thereby altering its ability to be activated by the ligands Delta and Serrate. This results in an activation of the Notch pathway at the equator of the eye[32]. Notch activation is thought to lead to the induction of the "PCP signal" at the presumptive equator. One such Notch-activated polarity signal is Four-jointed (Fj), a transmembrane protein, some of which is cleaved to produce a secreted fragment[33]. Fj is expressed at the midline from very early on, and a gradient of Fj can polarize ommatidia[34]. However, even in the complete absence of Fj, planar polarity decisions are still made with very high precision (0.3% errors). Thus, there must be some additional signal, commonly known as "factor X", that acts together with Fj to provide polarity information.

## Reading and Responding to the PCP Signal

The unknown polarity signal (Fj + factor X) is interpreted by the ommatidial precluster, dictating that the more equatorially located member of the R3/R4 precursors becomes R3, and directing the subsequent rotation of the cluster (Box 4, Fig. E). Much evidence has implicated communication by the Notch pathway between the R3 and R4 cell as being central to the decision to turn away from or towards the equator[35,36]. The member of the R3/R4 pair with higher Notch activity will take on the R4 fate, and the cluster will turn in a way that is coincident with this decision. Fz affects Notch activity in the R4 cell by enhancing Delta transcription in the R3 cell. (This is different, perhaps, than in the wing, where transcriptional input may not be necessary — in the eye, the Fz pathway feeds through the Notch pathway before the cells can properly interpret the PCP signal.) A number of genes that affect planar polarity in the eye may contribute to the appropriate activity of Notch in the R3/4 cells. *Sple*$^{1}$ is an allele of the LIM domain protein encoded by *pk-sple* [37], and in *sple*$^{1}$ mutants, Notch activity is inappropriately high in the R3 cell[38]. E(spl) proteins are transcriptional inhibitors that can repress proneural gene expression and are induced by Notch activity. Loss of Barbu, a gene homologous to E(spl)m4, results in dramatic planar polarity defects, which are probably due to a lack of appropriate Notch activity[39].

RhoA is important for the control of planar polarity in the eye as well as the wing. Loss of RhoA results in misrotated ommatidia as well as ommatidia that lack photoreceptors[16]. One way this can be easily imagined to work is that RhoA is important for the remodeling of the cytoskeleton that occurs as ommatidia rotate to their final position. That this occurs subsequent to Fz-Dsh signal is supported by the observation that reduction of RhoA can suppress a planar polarity phenotype that is induced by ectopic expression of Dsh or Fz.

## Involvement of the JNK Pathway

However, another model is suggested by studies that examined the effects of overexpression of a number of genes using the sevenless enhancer (sevE). SevE drives expression in the eye in subsets of differentiating cells after the furrow has passed. SevE-Fz and sevE-Dsh can both cause specific defects in planar polarity. Remarkably, driving RhoA or components of the JNK pathway in this manner not only can cause polarity defects but also can rescue *dsh*[1] mutant polarity phenotypes[40]. Based on these data, it has been proposed that RhoA acts to control transcription and thus to mediate the planar polarity signal by activating Jun[16,41,42,43]. Consistent with this, overexpressed Dsh in tissue culture can induce phosphorylation of Jun, while overexpression of a mutant Dsh that does not function in PCP signaling is impaired in its ability to phosphorylate Jun[40].

Misshapen (Msn), a Ste20-like kinase, is also needed for planar polarity in the eye and wing and for activation of JNK in tissue culture cells[44]. Loss of *msn* can block the polarity phenotype of *sevE-Dsh* and can block Dsh-induced phosphorylation of Jun in tissue culture. This suggests that Msn acts downstream of Dsh and upstream of Jun in the planar polarity pathway. TAK is a MAPKKK that may act in a JNK pathway, and ectopic expression of DN-TAK can induce polarity defects in the eye[42]. Analysis of loss of function of *tak* has not been done in the eye, so we cannot be sure that these are specific effects. Importantly, loss of Fz, Dsh, and Msn all induce polarity defects. However, loss of *jun* or its activating kinase, *bsk*, do not significantly affect polarity. This result could mean that there are redundant pathways that act in polarity, masking the *jun* and *bsk* phenotype. The p38 pathway has been suggested to be this redundant pathway[41]. However, it is also possible that *jun* and *bsk* are essential for the polarity phenotype that is caused by Dsh or Fz overexpression but not for the normal establishment of polarity. Several studies have linked activation of the JNK pathway in vertebrates with convergent extension and have suggested a common Fz->Rho->JNK pathway (see below). However, in mammalian systems, JNK is primarily activated by Cdc42 and Rac, and not by Rho[45]; yet in flies it is proposed that RhoA is acting through the JNK pathway. Furthermore, there is no evidence that Cdc42 has any effect on planar polarity in flies, and although Rac has been implicated in the control of planar polarity in flies, these data come from dominant-negative misexpression experiments[43]. Such experiments may be misleading because it is difficult to verify the specificity of such constructs.

## Many Pathways…

There are a large number of pathways that seem to feed into the determination of planar polarity in the eye, and it is not yet clear where they all act. Rasputin (Rin) is a G3BP (RasGAP-binding protein) that is needed for planar polarity in the eye and has been shown to interact genetically with RhoA[46]. Interestingly, Rin does not interact with sevE-Dsh or sevE-Fz and may lie in another pathway. Another “orphan” polarity gene is *expanded* (*ex*). *Ex* is a tumor suppressor gene with SH3 binding sites and an N-terminal 4.1 homology region. Loss of *ex* can also disrupt planar polarity, but *ex* does not seem to interact genetically with sevE-Fz and may also lie in a different pathway[47]. Another indication that planar polarity signaling acts in part through the control of transcription is that overexpression of a nuclear protein, poly-ADP-ribose polymerase (PARP), causes planar polarity defects in the eye, presumably by altering the transcription of important target genes[48]. However, loss-of-function analysis of PARP is required to be sure that PARP truly controls planar polarity and to determine its place in the pathway.

The JAK/STAT pathway also appears to participate in planar polarity in the eye[34], since both loss of function and ectopic expression of the ligand, unpaired (Upd), are sufficient to repolarize ommatidia. Upd is expressed at the midline (or at least the posterior midpoint of the eye). Although Upd may act through controlling *mirror* expression (since loss of Upd leads to an expansion of *mirror* expression), ectopic Upd can repolarize ommatidia at both the dorsal and ventral margins. Since *mirror* is not expressed in the ventral half of the eye, this indicates that Upd must have additional targets. Also, loss of downstream elements in the JAK/STAT pathway do not affect *mirror* expression yet do affect ommatidial polarity. Therefore the JAK/STAT pathway must also have additional, potentially more downstream targets.

In addition, there are several “core” components of the polarity pathway that act both in the wing and in the eye but have been explored more thoroughly in the wing than in the eye. Fmi/Stan, the seven-transmembrane cell adhesion molecule, has been shown to be crucial to planar polarity in the wing, where it localizes to proximal and distal cell boundaries. In the eye, *Fmi/Stan* is needed for planar polarity[12], but its localization in the eye is not yet known. Similarly, Dgo, an ankyrin repeat protein, is necessary for normal polarity in the eye and wing[13]. In the eye, loss of *Dgo* results primarily in achiral ommatidia, with weak rotation defects. By analogy with the wing, it is likely that Dgo is stabilizing Fmi/Stan and marking a distinct cell membrane. Similarly, the atypical cadherins, Fat and Dachsous, have been shown to be essential for planar polarity in the wing[49], but their phenotype in the eye has not yet been reported.

Strabismus (Stbm), also known as Van Gogh (Vang)[50], is a transmembrane protein that originally was identified due to its role in controlling planar polarity in the eye[51], where it is thought to be important in the R4 cell fate. Genetic data from experiments in the wing suggest that Stbm/Vang is essential for Fz signaling[50]. However, recent analysis in fish and frogs has identified Stbm as a Dsh-binding protein that suppresses Dsh’s ability to activate β-catenin[52].

### Controlling the Extent of Rotation

Genetic studies have indicated that once ommatidial rotation has started, the degree of rotation is also highly controlled[53]. In flies that are mutant for the MAPK-related Nemo (Nmo), ommatidia will initiate rotation in the correct direction. However, ommatidia lacking Nmo only rotate 45° rather than 90°. This suggests that Nmo is necessary to either initiate the last 45° turn or to block a stop signal that occurs at 45°. Roulette (Rlt), on the other hand, appears to tell ommatidia when to stop rotating. In the absence of Rlt function, ommatidia will rotate to variable degrees, often overshooting the normal 90° rotation. It is not yet clear how Nmo and Rlt function to control the degree of ommatidial rotation.

## PLANAR CELL POLARITY IN BRISTLES

Microchaetae, the small sensory bristles on the notum, or dorsal thorax, also display planar polarity. These bristles arise from a single precursor cell, called pI, that undergoes a series of stereotypical divisions to produce five cells[54,55] (see Box 5). PI division is oriented along the anterior–posterior (A–P) axis, and the orientations of the subsequent cell divisions follow from the pI axis. Numb and PON form an anterior crescent in the pI cell and are thereby asymmetrically segregated into the anterior daughter cell after division. In *Fz* and *Dsh*$^1$ mutants, the pI axis of division remains within the plane of the epithelium but is randomized with respect to the A–P axis[55]. If Fz signaling is lacking, Numb still forms a crescent, but it is not strictly anterior, and the spindle is no longer aligned with the A–P axis[55,56,57]. Since the Numb crescent still forms, this indicates that the cell does become polarized, but the axis is randomized. Fmi/Stan function is also needed for correct orientation of the spindle in pI[14].

How does Fz signaling lead to coordination of orientation of the spindle and the Numb crescent? Work in neuroblasts has demonstrated that apical–basal polarity in epithelial cells is essential for the subsequent polarity of the neuroblast (reviewed in [58]). An attractive idea is that planar polarity, too, is not created *de novo* but instead builds on already established apical–basal cell polarity. This proposal is supported by a recent analysis of how proteins essential for apical–basal polarity in epithelial cells are reorganized in bristle precursor cells in an Fz-dependent manner[56].

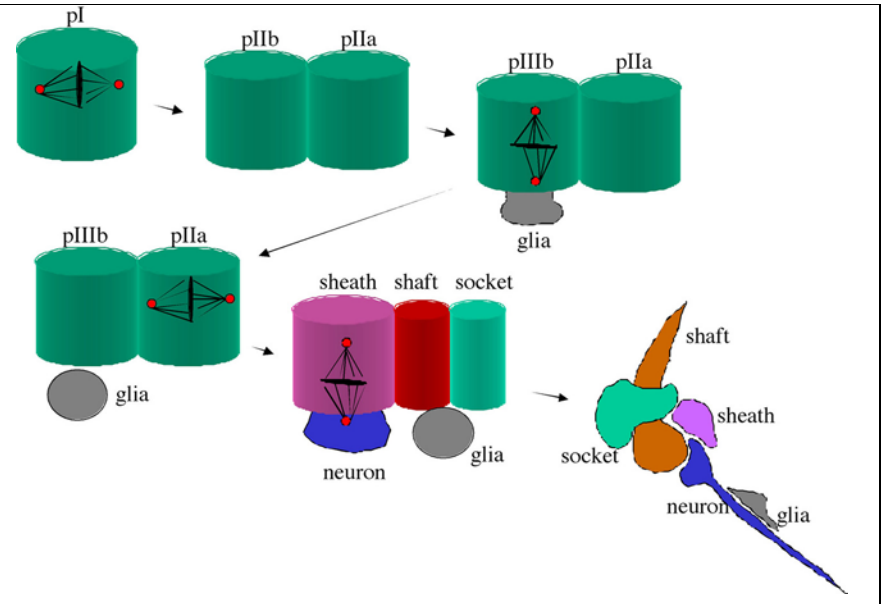

**BOX 5. Genesis of bristle polarity. FIG. F.** The bristle precursor cell pI divides within the plane of the epithelium, producing an anterior pIIb and a posterior pIIa. PIIb then divides perpendicular to the plane of the epithelium, producing a subepithelial glial cell that migrates away from the nascent bristle along an axon, and a pIIIb cell. PIIa then divides along an axis parallel to the pI division, giving rise to an anterior trichogen (shaft) and a posterior tormogen (socket) cell. Finally, pIIIb divides perpendicular to the plane of the epithelium, producing a basal neuron and an apical thecogen (sheath) cell[54,55]. The figure schematizes these divisions. Anterior is left, and apical is top.

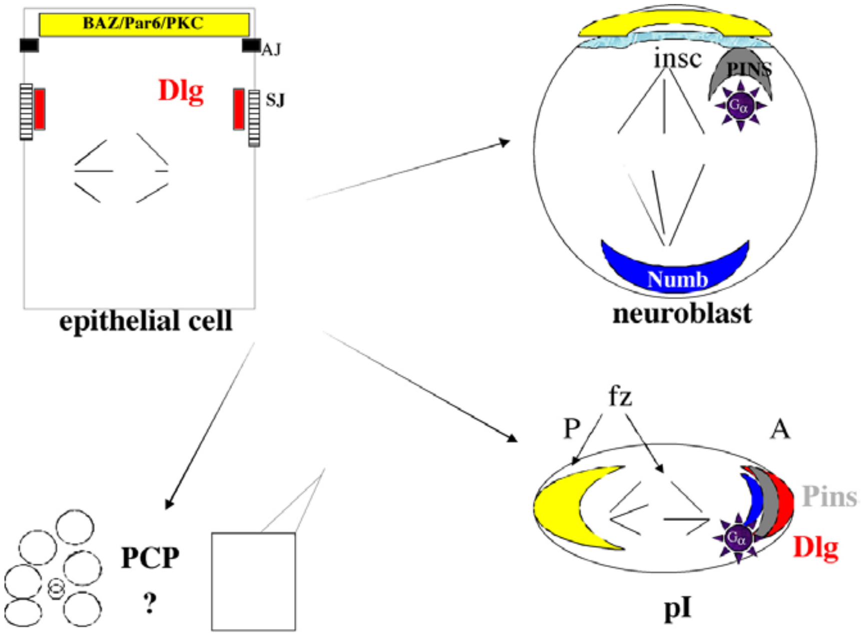

**FIG. G.** Polarity in epithelial cells, neuroblasts, and pI cells. Recent work has shown that many components that determine apical–basal polarity in epithelial cells are essential for the subsequent asymmetric division of neuroblasts and pI. In epithelial cells, the Baz/aPKC/Par6 complex (yellow) is apical to the adherens junction (AJ), while Dlg (red) is localized to septate junctions (SJ). As cells delaminate from the epithelium and become neuroblasts, they begin to express Insc (light blue). Baz recruits Insc to the apical cortex, and the Baz/Insc complex in turn recruits Pins (grey), which binds Gαi/o proteins (purple) and may activate G protein signaling. This complex, which has been shown to orient the neuroblast mitotic spindle and to regulate the basal localization of Numb (dark blue), is restricted to the basal cortex. In contrast, in pI cells, Insc is not expressed, and Pins becomes localized to the anterior cortex. The Baz/Par6/PKC complex relocalizes to the posterior cortex, while Numb colocalizes with Pins and Dlg. For clarity, only a few of the many asymmetrically distributed proteins are shown here. For a more detailed treatment of epithelial cells, see reference [3]; for more extensive treatment of neuroblasts, see reference [58].

A key component of the establishment of epithelial cell polarity is the multiple-PDZ protein, Bazooka (Baz)[59]. Baz forms a protein complex with Par-6 and atypical protein kinase C (aPKC) and is localized apically, just above the adherens junction in epithelial cells[60] (see Box 5). In pI, initial Baz localization is apical to the adherens junction, where it is found uniformly distributed, as it is in epithelial cells[56]. But during mitosis, Baz and aPKC begin to accumulate at the posterior cortex, opposite to Numb. While Pins, (which binds Gαi/o protein and can activate G protein signaling) is now found at the anterior cortex with Numb.

In epithelial cells, two other proteins that are crucial for apical–basal polarity are Discs large (Dlg) and Scribble (Scrib). Dlg and Scrib localize in epithelial cells to the septate junction, a structure found below the adherens junctions. Dlg contains 3PDZ domains, an SH3 domain, and a guanylate kinase domain, while Scrib is a LAP protein that contains leucine-rich repeats and PDZ domains[61,62]. These proteins are also reorganized in the pI cell as it undergoes mitosis. As in epithelial cells, Dlg is symmetric below the adherens junction early, but then in the dividing pI cell, both Dlg and Pins become enriched at the anterior cortex with Numb. Genetic analysis indicates that both Pins and Fz are necessary to localize Baz to the posterior, and that Baz, Dlg, and Pins are needed to localize Numb to the anterior cortex. Taken together, these results suggest that Fz plays a role in orienting the division along the A–P axis and ensures that coordination of the Numb complex with the spindle produces an asymmetric division.

All cells that later develop planar polarity initially possess apical–basal polarity. This beautiful example of how *Fz* signaling acts to reorganize apical–basal cell polarity to control planar polarity may provide a model for how Fz may generally act to remodel the cytoskeleton in planar polarity. However, it is worth noting that in *Fz* mutants, the pI axes are randomly oriented in the medial microchaetae, but in the adult, these bristles are little affected, compared to the lateral microchaetae. This suggests another layer of control for the determination of planar polarity in the bristles and is consistent with the appearance of several apparently redundant systems that control planar polarity in the eye.

## CONSERVATION OF THE PCP SIGNALING MECHANISM

Here, we summarize what is currently known about the signals driving convergent extension movements and the planar polarization of cochlear hair cells, and we discuss the likelihood that these signals are homologous to the *Drosophila* PCP signal.

### Convergent Extension

During vertebrate convergent extension (CE), elongation of the A–P axis and a concomitant decrease in the mediolateral dimension are driven by cellular elongation in the mediolateral axis and intercalation of cells (see Box 6). Some time ago, it was noted that a dominant-negative form of Dsh interferes with *Xenopus* CE, raising the possibility that either a canonical Wnt, or a PCP-like mechanism, is responsible for these cell movements[63]. Recently, Wnt11 was identified in both *Xenopus* and zebrafish because of its requirement in CE[64,65]. In both studies, evidence was presented indicating that Wnt11 activates a non-canonical pathway that resembles the PCP signaling pathway.

**BOX 6. Convergent extension movements during gastrulation.** Mesodermal cells prior to convergent extension are roughly symmetric. Upon induction of convergent extension, the cells elongate along an axis orthogonal to the A–P axis and begin to migrate with respect to each other, resulting in intercalation. This movement produces a lengthening of the A–P axis. Neuroectodermal cells undergo a similar movement, although their elongation and movement are unipolar rather than bipolar[73].

Tools to distinguish whether the CE signal resembles canonical or noncanonical Wnt signaling derive from the analysis of *Drosophila* Dsh deletion constructs. Several studies demonstrated a differential requirement for Dsh domains in PCP and canonical Wg signaling[40,66]. Furthermore, localization of Dsh to the cell cortex is a necessary step in PCP but not canonical Wg signaling[9,66]. Finally, elevated phosphorylation of Dsh has been proposed as a marker for both the PCP and the canonical Wnt signaling pathways[9,67].

These differences in Dsh requirements and behavior have been exploited to make inferences about whether the Wnt-mediated CE processes in *Xenopus* and zebrafish reflect the activity of the canonical or a PCP-like pathway. Dominant-negative Wnt11 (DN-Wnt11) blocks *Xenopus* CE, and this can be rescued by either full-length *Xenopus* Dsh (Xdsh) or by truncated forms of Xdsh much like some that retain function in the *Drosophila* PCP but not canonical Wg signaling pathway[65]. In contrast, the *DN-Wnt11* phenotype was not rescued by activators of the canonical Wnt pathway, such as activated β-catenin. Similar results were obtained in the zebrafish study, in which the *silberblick (slb)/Wnt11* mutant displayed a CE defect that could be rescued selectively by Dsh constructs similar to those that activate the PCP pathway, but not by activation of canonical Wnt signaling[64]. Furthermore, a Dsh construct selectively dominant negative for PCP signaling blocks CE in wild-type zebrafish. These studies demonstrate that a noncanonical Wnt pathway activates CE, and they imply, by virtue of the similar domain requirements for Dsh, that the pathway is similar to the PCP pathway. A similar study indicates that Xfz7 acts as a receptor for this pathway[68]. A survey of the ability of various Fz proteins to localize Xdsh to the cell cortex, and of various Dsh-deletion constructs to localize to the cortex, demonstrates that canonical Wnt axis-inducing activity does not segregate with Dsh cortical localization. Similarly, Dsh hyperphosphorylation does not correlate with canonical Wnt signaling activity. This again suggests that a noncanonical PCP-like pathway is associated with these phenomena[69].

Evidence has been presented that this PCP-like CE-activating pathway in *Xenopus* induces planar cell polarity in migrating mesodermal cells. In wild-type *Xenopus*, these cells elongate along the mediolateral body axis, and they form and maintain lamellipodia preferentially along the same axis[70]. These planar asymmetries are abolished by expression of a dominant-negative Xdsh that does not interfere with canonical Wnt signaling, and by overexpression of full-length Xdsh. In contrast, inhibiting the canonical Wnt pathway does not affect these planar asymmetries. Furthermore, association of Xdsh with the cell cortex is first observed in cells undergoing CE but not in cells prior to initiation of CE. It is also seen in explanted cells that are stimulated to undergo CE, but not in naïve explants. Therefore, this pathway polarizes cells in the plane of the epithelium, as does the *Drosophila* PCP pathway.

These studies, taken together, indicate that a noncanonical Wnt pathway regulates CE. However, the inference that this pathway resembles the PCP pathway depends solely on the behavior of Dsh and Dsh derivatives. The argument recently has been strengthened by the findings that vertebrate homologs of another *Drosophila* PCP gene are active in CE. Two studies examining *Xenopus*, zebrafish, and mouse Stbm/Vang demonstrate that Stbm is a regulator of CE. Loss of Stbm/Vang function in frog and fish embryos produces a failure of axial elongation consistent with a defect in CE[71], and the Ltap mouse mutation, which produces neural tube closure defects, maps to a mouse homolog of Stbm/Vang[72]. Since the CE signal affects both mesoderm and neural ectoderm, this phenotype is consistent with a defect in CE[73]. Another study has shown that induced loss and gain of function of the Fat-related paraxial protocadherin inhibits CE[74]. *Drosophila* Fat is implicated in PCP signaling[49,75], though its precise role has not been reported. Although these studies do not fully document the apparent CE phenotypes, they add significant weight to the hypothesis that CE signaling is functionally and mechanistically homologous to *Drosophila* PCP signaling.

Because *Drosophila* Dsh has been shown to activate JNK signaling in cell culture and *in vivo*[40], JNK activation has been used as a surrogate assay for CE signaling activity in vertebrate systems. While correlations between CE activity and JNK activation are observed in these studies, no causal relationship has been demonstrated.

Three proteins appear to toggle Dsh function between canonical Wnt signaling and CE signaling. Casein Kinase I (CKI) activity is required for canonical Wnt signaling and may function by phosphorylation of Dsh[76,77]. Inhibition of CKI activity also interferes with CE in *Xenopus* and activates JNK signaling in cultured cells[77]. Similarly, *Xenopus* and zebrafish Stbm bind Dsh and inhibit canonical Wnt signaling. Stbm overexpression also modulates CE, and this correlates with an ability to activate JNK signaling in cell culture. Finally, *Drosophila* Naked Cuticle (Nkd) is a feedback inhibitor of canonical Wnt signaling that functions by binding and antagonizing Dsh[78]. Mammalian Nkd, like the *Drosophila* homolog, binds Dsh, and overexpression antagonizes its function in canonical Wnt signaling. Yan et al.[79] have shown that overexpression of mammalian Nkd blocks *Xenopus* CE. Overexpression of mammalian Nkd also activates JNK signaling in cell culture, and this correlation has been interpreted as evidence that Nkd stimulates the PCP-like/CE function of Dsh. Interestingly, however, overexpression of *Drosophila* Nkd induces disruption of PCP signaling, presumably by binding Dsh, but the lack of a loss-of-function phenotype for *Nkd* in PCP signaling argues against a role in normal PCP signaling.

Taken together, these data indicate that JNK signaling is activated under circumstances in which CE signaling is also activated. What is puzzling, however, is that Nkd, despite its apparent lack of a role in PCP signaling, seems to activate JNK signaling when overexpressed. It remains to be determined how and why switching off Dsh function in canonical Wnt signaling correlates with activating its JNK-activating and CE-like/PCP signaling functions.

At least one observation from the vertebrate studies may be informative for understanding the fly PCP signaling mechanism. It has been inferred that a Wnt serves as a ligand that provides directional information during PCP signaling in the fly, despite a lack of direct evidence. Transplant experiments demonstrated that zebrafish Slb/Wnt11 activity is required in the paraxial mesoderm to induce CE in axial mesoderm[64]. However, rescue of CE can be achieved by injection of *Wnt11* mRNA into one-cell embryos, suggesting that localized activity of the Wnt is not required for CE. It might therefore be the case that *Drosophila* PCP also depends on the permissive activity of a Wnt, but that the Wnt does not carry directional information.

## Polarity of Cochlear Hair Cells

Hair cells in the inner ear display a remarkable polarization of the stereocilia, forming a step-like pattern that is strikingly reminiscent of planar polarity in the fly (see [4]). This organization is very highly controlled, with position in the ear precisely correlating with both the size and the orientation of stereocilia (reviewed in [80]; see Box 7). However, although morphology suggests that these are analogous systems, as yet there are insufficient data to demonstrate that they are controlled by similar pathways. To determine if these are truly analogous systems, it will be important to see if defects in Fz or Dsh disrupt hair-cell formation. Interestingly, Frizzled4 is expressed in the inner ear, and loss of Fz4 causes deafness and progressive loss of hair cells[81].

Our understanding of the development of hair cells is rapidly growing. This is due to the fact that disruption of stereocilia leads to deafness and balance problems. More than 50 loci have been implicated in human deafness, and studies of families with syndromic and nonsyndromic deafness, in conjunction with analysis of mouse models of deafness, have recently uncovered a number of genes involved in stereocilia formation and maintenance (see Box 7). Genes that have been identified in deafness syndromes demonstrate the importance of various adhesion molecules, transcription factors, cytoskeletal elements, and cytoskeleton-associated proteins. Strikingly, defects in myosin VIIa cause defects in wing hair-cell polarity in *Drosophila*[17] and cause defects in stereocilia formation in inner-ear hair cells[82]. Other myosins such as myosin XV also cause defects in stereocilia formation, with abnormally short stereocilia that have supernumerary rows[83]. These defects are not yet linked to PCP signaling.

One would imagine that the ideal planar polarity phenotype in the ear would be one in which stereocilia form properly but they are misaligned. Such a phenotype will, of course, only be discernable if the same molecules are not also used in constructing the ear or determining hair-cell fate. The development of *in vitro* culture of inner ear, and immortalized hair-cell lines, opens the possibility of further analysis[84]. It will be interesting to see what role, if any, the genes that have been implicated in stereocilia formation have in determination of planar polarity in *Drosophila* eye and wing.

**BOX 7. Polarity of cochlear hair cells (FIG. H).** (A) Development of hair cell in chick inner ear. (Adapted from Tilney et al.[80], with permission from the *Annual Review of Cell and Developmental Biology*, Vol. 8, ©1992 by Annual Reviews. www.AnnualReviews.org.) (B) Mature chick hair cell. (Reproduced from *The Journal of Cell Biology*, 1991, 112(4), 641 by copyright permission of The Rockefeller University Press.)

The development of hair-cell polarity has been extensively characterized in beautiful studies of the chick cochlea (for review see [80]). Initially, small actin-filled microvilli cover the apical surface of both hair cells and supporting cells. At about embryonic day eight (E8), the apical surface of hair cells begins to display thicker actin-filled structures called stereocilia, which are sparse and randomly distributed over the apical surface. Near the center of the apical surface is a single microtubule-filled kinocilium. At about E9, the entire apical surface is filled with stereocilia, and the kinocilium has moved to a random position at the periphery. Between E9 and E11, kinocilia move so that the kinocilia on all cells are now at the same side of the cell. The typical staircase pattern of stereocilia develops between E10 and E12. First, stereocilia that are closest to the kinocilium begin to elongate, then those that are in the next row, and so forth. As the hair-cell surface grows, new stereocilia form. Later, stereocilia far from the kinocilium are reabsorbed. As the staircase forms, the stereocilia bundles also rearrange, changing from a round bundle to a semicircular one, and finally to the rectangular adult shape. In the final form, stereocilia are connected by tip links and ankle links. Tip links begin to form as the staircase forms and are thought to be involved in stabilizing the stereocilia. Tip links have an additional function in transforming shear between the stereocilia to tension, which is then converted via channels located near the links to action potentials. The tip links therefore are also an integral part of the signal transduction apparatus for hearing.

Mutations in genes homologous to PCP genes have been identified in congenital deafness loci. One particularly well-studied human condition is Usher syndrome. Usher syndrome is a homogeneous, autosomal recessive condition marked by deafness, vestibular dysfunction, and blindness due to progressive retinitis pigmentosa[111]. A number of different Usher loci have been cloned, and many have provided insight into the control of stereocilia in hair cells. Ush1b encodes myosin VIIa, and analysis of patients with Ush1b mutations indicated that they have severe degeneration of the organ of Corti[112]. Insights into the function of myosin VIIa in the development of stereocilia have come from analysis of mouse and zebrafish mutants, which show dispersion of the normally regular hair-cell stereocilia into irregular clumps across the apical cell surface[82,113]. Since myosin VIIa is localized to the "ankle" region of the stereocilia, it has been suggested that it is the intracellular attachment to the cytoskeleton for these extracellular links[114]. Interestingly, a myosin VIIa mutant in *Drosophila* also reveals a role in planar polarity determination in the wing[17]. Mutations in myosins VI and XV also cause hearing loss in man and/or mouse. Analysis of the shaker2 mouse, which is mutant for myosin XV, indicates that without myosin XV, inner hair cells have very short stereocilia organized in supernumerary rows with no tip links apparent. Myosin VI and VIIa localize to the vesicle-rich pericuticular necklace, thought to be a region for membrane trafficking[114].

Usher syndrome type 1D is caused by mutation of Cdh23[115,116]. Cdh23 is an extremely large cadherin, with homologies to *Drosophila* Fat, and is one of a number of large adhesion molecules that have been implicated in deafness and shown to affect stereocilia. Within the cochlea, Cdh23 expression is restricted to the hair cells, and it has been suggested that it may function to cross-link stereocilia. Cadherin 23 is mutated in the Waltzer mouse, causing circling behavior and hearing loss[117,118]. Studies of mouse mutants have allowed analysis of the role of this cadherin in stereocilia function. Another large cadherin, Cdh15, is mutated in Ush1F patients[119,120]. Analysis of mice with mutations in Cdh15 (the Ames Waltzer mouse) revealed hair-cell defects such as splayed stereocilia[119].

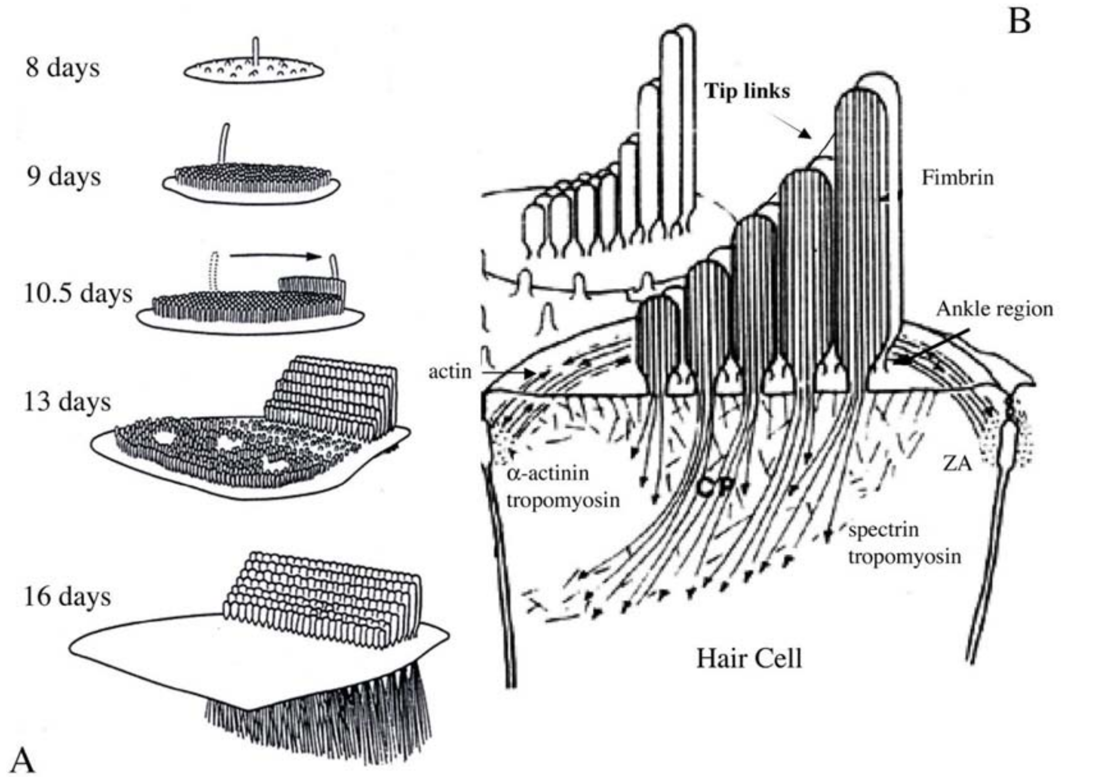

Since the core of the stereocilium is composed of cross-linked actin filaments, one would expect that mutations in actin-binding proteins might cause human deafness. Some actin bundling proteins (such as Epsin) have been shown to be involved in stereocilia maintenance and hearing in the mouse[121]. Mutations in a formin-like protein, Diaphanous, result in human hearing loss[122], but since a mouse mutation is not known, how Diaphanous affects the development or maintenance of stereocilia is unknown. Interestingly, in *Drosophila*, Diaphanous has been shown to be necessary for the recruitment of myosin to the membrane[123], and it will be interesting to see if myosins are mislocalized in stereocilia that lack Diaphanous. Similarly, Ush1c has been shown to encode Harmonin, a PDZ-domain-containing protein[124], but analysis of the role of Harmonin in stereocilia development awaits a mouse knockout.

A number of proteins involved in cell–cell and cell–substratum interactions have been associated with deafness. Mutations in the $\alpha 8$ integrin cause deafness, and analysis of a mouse with a targeted deletion in $\alpha 8$ integrin shows fused and collapsed stereocilia[125]. $\alpha 8\beta 1$ integrin, as well as its ligand fibronectin and FAK, localizes to the apical surface when stereocilia are forming, suggesting that it may have a role in directing stereocilia formation. Components of the extracellular matrix such as collagen have also been linked to human deafness, though this is often due to gross morphological defects of the ear rather than to disruptions in the structure or alignment of stereocilia.

Hair cells have distinct structural requirements, such that one might expect that specific transcription factors are important for coordinating a "hair-cell developmental program". One candidate transcription factor is POU4F3, a POU-domain transcription factor. Mutations in POU4F3 result in progressive deafness in humans. In a mouse knockout, there are no hair cells in the adult. However, examination of embryonic stages revealed that hair cells were originally present, but they were disorganized[126]. These immature hair cells contain many of the components of stereocilia, such as myosin VI and VIIa, and may only need a few key POU4F3 target genes to assemble the mature stereocilia.

Between the extensive EM studies of hair-cell development in model organisms and the rapidly expanding list of genes that have been implicated in hair-cell development due to human deafness, significant progress has been made in understanding the role of the cytoskeletal and cell surface molecules in organizing stereocilia.

## CONCLUSIONS

At present, we still do not know what the signal is that sets up long-range planar polarity in any system. Analysis in the *Drosophila* wing and eye has revealed a conserved signaling pathway downstream of an unknown signal. The core components of this pathway consist of a Fz/Dsh signaling complex whose recruitment depends on Fmi/Stan and whose stabilization depends on cytoskeletal proteins such as Dgo. There are many questions remaining as to how the Fz/Dsh signal is implemented into a remodeling of the cytoskeleton and the cell surface to give the final planar polarized structure. Analysis of the asymmetric division of the pI bristle precursor cell has indicated that Fz signaling may act to remodel apical–basal polarity by acting on cortical proteins such as Bazooka. Vertebrate planar polarity may be evident in convergent extension in *Xenopus* and zebrafish, where the Fz/Dsh/Stbm signaling system seems to play analogous roles. Inner hair-cell polarity in the vertebrate inner ear has striking planar polarization, and our understanding of how cell surface and cytoskeletal proteins are reorganized seems strongest there; but as yet we have little evidence that these disparate systems are controlled by the same genetic network. If the organization of stereocilia is truly analogous to planar polarity in the fly eye and wing, insights from these divergent systems may well allow rapid progress in our understanding of how cells and tissues become organized within the plane of the epithelium.
